# Evaluation of bone mineral density in patients with gestational diabetes mellitus by ultrasonic bone mineral density measurement combined with Vitamin-D deficiency and analysis of influencing factors

**DOI:** 10.12669/pjms.38.4.5090

**Published:** 2022

**Authors:** Lulu Han, Jingjing Ma, Shenghai Wang, Zhihong Li

**Affiliations:** 1 Lulu Han, Department of Endocrinology, Baoding First Central Hospital, Baoding, Hebei, 071000, China; 2 Jingjing Ma, Department of Endocrinology, Baoding First Central Hospital, Baoding, Hebei, 071000, China; 3 Shenghai Wang, Intensive Care Unit, Affiliated Hospital of Hebei University, Baoding, Hebei, 071000, China; 4 Zhihong Li, Department of Endocrinology, Baoding First Central Hospital, Baoding, Hebei, 071000, China

**Keywords:** Gestational diabetes mellitus, Triacylglycerol, Vitamin-D, Ultrasonic bone mineral density

## Abstract

**Objectives:**

To investigate the evaluation of bone mineral density (BMD) in patients with gestational diabetes mellitus (GDM) by ultrasonic bone mineral density measurement combined with Vitamin-D deficiency and its influencing factors.

**Methods:**

A total of 100 patients with gestational diabetes mellitus (GDM) admitted to our hospital from January 2017 to December 2020 were selected as the GDM group, and another 100 pregnant volunteers who underwent physical examination in our hospital were selected as the healthy control group. The levels of triacylglycerol (TG), total cholesterol (TC), low density lipoprotein cholesterol (LDL-C), glycated hemoglobin (HbA1c), fasting insulin (FINS), C-peptide, procollagen, parathyroid hormone (PTH), serum calcium, 25-hydroxyvitamin-D3 (25-OH-D) and ultrasonic BMD Z-score were determined and compared between the two groups. Moreover, the effects of different levels of 25-OH-D on the above indexes were compared, and multivariate logistic regression analysis was performed.

**Results:**

Compared with the healthy reference group, the levels of TG, TC, LDL-C, PTH, HbA1c, FINS and C-peptide of GDM group were significantly increased (P < 0.05), while BMD Z-score and 25-OH-D levels were significantly decreased. In the GDM group, the lower the 25-OH-D levels, the higher the FPG, HbA1c and FINS levels and the lower the serum calcium level and BMD Z-score, with statistically significant differences (P < 0.05). Multivariate logistic regression analysis showed that 25-OH-D was an independent risk factor affecting BMD in patients with GDM (P<0.05).

**Conclusions:**

Patients with GDM have lower BMD and 25-hydroxyVitamin-D3 levels. Measurement of BMD and 25-hydroxyVitamin-D3 levels can help assess the progression of GDM. BMD has a close bearing on Vitamin-D deficiency in patients with GDM.

## INTRODUCTION

Gestational diabetes mellitus (GDM) is a type of impaired glucose tolerance with varying degrees that occurs during gestation period or first occurs during pregnancy.^1^ GDM is a common metabolic disorder during gestation period, which seriously threatens the health of pregnant women and newborns. Pregnant women with GDM often have lumbago in different parts and degrees, which may be related to abnormal bone metabolism caused by abnormal glucose metabolism.^2^ During pregnancy, women’s hormone levels change significantly, and their needs for various micronutrients gradually increase, making them vulnerable to Vitamin-D deficiency.^3^ In the third trimester of gestation, a large amount of Vitamin-D is needed owing to the rapid development of the fetus.^4^ Pregnant women who cannot supplement Vitamin-D in time may suffer from numerous adverse effects, such as Vitamin-D deficiency, bone loss and bone density reduction, which in turn affects the related immune response.^5^ Vitamin-D is an essential substance for glucose to stimulate insulin secretion and maintain normal glucose tolerance under physiological conditions, and insulin resistance is an important cause of diabetes.^6^ Therefore, monitoring Vitamin-D level is crucial for GDM patients.

Parathyroid hormone (PTH) reflects the activity of osteoclasts, with its main function to regulate the metabolism of calcium and phosphorus inside body, increase blood calcium and reduce blood phosphorus, while Vitamin-D promotes the utilization of calcium and induces the secretion of PTH.^7^ Ultrasonic bone mineral density (BMD) is characterized by non-invasive, non-radioactive, simple and portable operation, and relatively little impact on the fetus and mother. BMD has certain advantages in reflecting bone microstructure.^8^ Up to now, few studies have been conducted on the correlation between bone metabolism indexes other than Vitamin-D, such as PTH and BMD, and GDM. In this study, ultrasonic BMD measurement and the detection of bone metabolism indexes such as Vitamin-D were carried out on patients with GDM to investigate whether Vitamin-D deficiency affects blood glucose and insulin levels, aiming to explore whether GDM has an impact on bone metabolism and BMD.

## METHODS

A total of 100 patients admitted to our hospital due to GDM from January 2017 to December 2020 were selected. All the pregnant women were 20-39 years old, and all were single pregnancy. The gestational age of the fetus ranged from 24-35 weeks. In addition, 100 pregnant volunteers who underwent antenatal examination in our hospital were selected as the healthy control group, ranging in age from 22-38 years old. The gestational age of the fetus ranged from 24-35 weeks. The comparison of the two groups of basic data showed no statistical difference and was comparable. All patients and their families in this study were informed and consented, and approved by the relevant medical ethics committee.

### Ethical approval:

The study was approved by the Institutional Ethics Committee of Baoding First Central Hospital on November 18, 2019(No.2019064), and written informed consent was obtained from all participants

### Inclusion Criteria:

- Patients who meet the diagnosis of GDM, which is defined as normal glucose metabolism before pregnancy, and diabetes diagnosed during pregnancy;^9^
- Patients without bad habits such as smoking and drinking during pregnancy;
- Patients with normal mental status and ordinary understanding and cognitive abilities;
- Patients with complete personal data and blood glucose examination records.

### Exclusion Criteria:

- Pregnant women with essential hypertension, diabetes, polycystic ovary syndrome, heart disease and severe liver and kidney dysfunction
- Premature infants or macrosomia;
- Patients taking Vitamin-D and drugs affecting bone metabolism in the last 4 weeks;
- Patients with previous bone metabolic diseases;
- Patients who did not agree to be enrolled in this study.

Five ml of venous blood was collected from all patients on an empty stomach in the morning, and the venous blood was centrifuged in a serum centrifuge at a speed of 3000r/min for 10min, and then the serum was removed. A Cobas e411 automatic immune analyzer manufactured by Roche and a Roche special kit were utilized to detect the levels of serum PTH and blood calcium.

An automatic glycosylated hemoglobin analyzer was used to monitor the level of glycosylated hemoglobin (HbA1c), and a Cobas e602 automatic electrochemiluminescence immunoassay analyzer manufactured by Roche was used to monitor the levels of FINS and C-peptide.

A Norman luminescence analyzer was used to detect 25hydroxyVitamin-D3 by chemiluminescence immunoassay. Japanese Furuno CM-200 ultrasonic BMD instrument was used, and the detection result was similar to that of DXA lumbar BMD. BMD Z-scores of right foot calcaneal bone in the two groups were measured and recorded for 3 times, and the average value was taken. Serum calcium concentration was measured by a semi-automatic analyzer. Osteoporosis was defined as the BMD Z-scores below -2.0. All patients with GDM were differentiated, among which 48 patients with 25-hydroxyVitamin-D3 lower than 20ug/L were included in the Vitamin-D deficiency group, 32 patients with 25-hydroxyVitamin-D3 higher than 20ug/L and lower than 30ug/L were included in the Vitamin-D insufficiency group, while 20 patients with 25-hydroxyVitamin-D3 higher than 30ug/L were included in the normal Vitamin-D group.

### Statistical Analysis:

All data in this study were analyzed and processed by SPSS 22.0 software, and the counting data were expressed represented by %. Inter-group comparison was performed by *X^2^* test, the measurement data were expressed in (x̄ ± s), and inter-group comparison was tested by LSD-t. P<0.05 indicates a statistically significant difference.

## RESULTS

The pregnant women of GDM group were 20-39 years old, with an average age of (30.2 ± 3.5) years old. The gestational age of the fetus ranged from 24-35 weeks, with a mean gestational age of (29.6 ± 5.2) weeks. The pregnant volunteers of the control group were 22-38 years old, with an average age of (28.6 ± 2.5) years old. The gestational age of the fetus ranged from 24-35 weeks, with a mean gestational age of (29.3 ± 5.6) week. The comparison of the two groups of basic data showed no statistical difference (P>0.05). The levels of TG, TC and LDL-C in the GDM group were higher than those in the healthy control group, with statistical differences (P<0.05). Table-I. The levels of PTH in the GDM group were higher than those in the healthy control group (P < 0.05), with no significant difference in serum calcium (P > 0.05). Table-II.

**Table-I:** Analysis of the levels of TG, TC and LDL-C between the two groups (x¯±s).

Group	Number of cases	TG (mmol/L)	TC (mmol/L)	LDL-C (mmol/L)
Healthy control group	100	1.36±0.17	3.59±0.43	3.14±0.32
GDM group	100	1.88±0.21	4.79±0.52	3.89±0.45
t		19.250	17.780	13.580
P		0.001	0.001	0.001

**Table-II:** Analysis of the levels of serum calcium and PTH between the two groups (x¯±s).

Group	Number of cases	Serum calcium (mmol/L)	PTH (pg/ml)
Healthy control group	100	2.24±0.35	40.36±8.54
GDM group	100	2.18±0.40	112.35±12.37
t		0.955	18.611
P		0.104	0.000

The levels of HbA1c, FINS and C-peptide in the two groups were compared and analyzed are shown in Table-III. The levels of HbA1c, FINS and C-peptide in the GDM group were higher than those in the healthy control group, with statistically significant differences (P < 0.05).Z-scores and 25-hydroxyVitamin-D3 levels were compared between the two groups. The Z-score and 25-hydroxyVitamin-D3 level in the GDM group were lower than those in the healthy control group, with statistically significant differences (P < 0.05). Table-IV BMD Z-score and serum calcium levels were lower, and insulin, blood glucose, and serum calcium levels were higher in the Vitamin-D deficiency group than those in the Vitamin-D insufficiency group. BMD Z-score and serum calcium levels were higher, while insulin, blood glucose and serum calcium levels were lower in the normal Vitamin-D group, with statistically significant differences between groups (P < 0.05).Table-V

**Table-III:** Analysis of the levels of HbA1c, FINS and C-peptide between the two groups (x¯±s).

Group	Number of cases	HbA1c (%)	FINS (μU/ml)	C peptide (ng/ml)
Healthy control group	100	4.23±0.45	16.34±1.53	1.43±0.22
GDM group	100	6.41±0.63	25.76±2.41	2.64±0.31
t		28.160	33.000	31.830
P		0.001	0.001	0.001

**Table-IV:** Comparison of BMD Z-score and 25-hydroxyVitamin-D3 level between the two groups (x¯±s).

Group	Number of cases	BMD Z-score	25-hydroxyVitamin-D3 (ug/L)
Healthy control group	100	-0.74±0.91	39.81±4.59
GDM group	100	-1.98±0.51	24.29±2.81
t		11.890	28.840
P		0.001	0.001

**Table-V:** Comparison of GDM-related levels of different Vitamin-D content.

Detection indexes	Vitamin-D deficiency group (n=48)	Vitamin-D insufficiency group (n=32)	Normal Vitamin-D group (n=20)
FINS (μU/ml)	17.34±1.53	14.34±1.53	12.34±1.53
BMD Z-score	-2.12±0.47	-2.01±0.41	-1.84±0.36
Blood glucose (mmol/L)	6.14±0.62	5.83±5.21	5.12±4.81
HbA1c (%)	7.24±0.71	5.85±0.63	5.12±0.45
Blood calcium (mmol/L)	1.84±1.46	2.03±0.19	2.26±0.21

Note: ^a^P<0.05 compared with the Vitamin-D deficiency group; ^b^P<0.05 compared with the Vitamin-D insufficiency group.

The occurrence of GDM was taken as the dependent variable, Z-score and 25-hydroxyVitamin-D3 were taken as independent variables, and these variables were included in the multivariate logistic regression model analysis. It was found that both Z-score and 25-hydroxyVitamin-D3 were the factors for osteoporosis in patients with GDM (P < 0.05). Table-VI

**Table-VI:** Multivariate Logistic regression analysis of osteoporosis in GDM.

Factor	Beta	SE	Wald	P value	OR value	95% CI
Z-score	1.536	0.852	13.275	0.001	2.326	1.342-4.631
D325-hydroxyVitamin-D3	1.758	0.724	11.756	0.001	4.856	1.827-9.135

## DISCUSSION

Pregnancy, as a more complicated physiological change, will change the blood circulation and immune system of pregnant women to a large extent. Pregnant women have varying degrees of bone loss, and abnormal glucose metabolism will also aggravate the loss of bone.^10^ GDM has an extremely high incidence among pregnant women,^11^ which usually occurs around 24-28 weeks of gestation, and most patients can recover after delivery.^12^ The average age of pregnant women in China is around 24, likewise exactly the peak bone mass period. For the purpose of ensuring the healthy growth and development of the fetus, pregnant women are in urgent need to adapt to their own physiology and make self-regulation to maintain a normal range of serum calcium concentration.^13^ There is still controversy about the time when bone mass appears in patients with GDM. Ultrasonic BMD measurement during pregnancy boasts a variety of benefits, such as reflecting the degree of bone calcium loss during pregnancy in an accurate, timely, and objective manner, so as to provide a basis for early nutritional intervention and calcium supplementation.

During early pregnancy, the increase in maternal fat deposits is facilitated by insulin, followed by increased adipose tissue breakdown and subsequent hypertriglyceridemia, mainly as a result of insulin resistance (IR) and estrogen effects.^14^ It was found in our study that the levels of TG, TC, LDL-C, FBG, FINS, C peptide and HbA1c in patients with GDM were significantly higher than those in healthy population. The results were similar to those of Sánchez-Vera et al.^15^ Studies have shown that abnormal glucose metabolism in pregnant women will aggravate bone loss.^16^ During pregnancy, the peak bone mass of pregnant women will be severely decreased, which may be attributed to the fact that the pregnant women need to provide calcium for the fetal bone development, resulting in a decrease in the pregnant women’s own bone mass.^17^ In our study, the BMD of the GDM group was lower than that of the healthy population, but there was no statistical difference in calcium content between the two groups, which does not exclude statistical error and is also related to the small sample size included in this study. Wang Q et al.^18^ found in their study that the BMD of pregnant women with GDM was significantly lower than that of pregnant women with normal blood glucose, and believed that prevention of GDM in pregnant women was crucial for their bone health, which was similar to the results of this study. We found that there was a negative correlation between serum 25-hydroxyVitamin-D, PTH and BMD in the GDM group which was similar to the research results of Kramer et al.^19^ This may be attributed to the metabolic environment in pregnant women, in order to meet the growth and development of the fetus, regulating the increase of parathyroid hormone secretion, increasing the number of osteoclasts, promoting the dissolution of bone salts, bone calcium into the blood to supply nutrients needed for fetal growth, leading to the reduction of bone mineral density.^20^ In the GDM group of our study, with the increase of 25-hydroxyVitamin-D level, the Vitamin-D deficiency group had higher levels of FPG, HbA1c and insulin, but lower levels of Ca and BMD compared with the Vitamin-D insufficiency group and the Vitamin-D normal group. Results from a systematic review published by Zhang et al.^21^ showed that blood Vitamin-D levels were negatively correlated with fasting blood glucose (FPG) and insulin resistance index, which are similar to the results of this study. Therefore, Vitamin-D supplementation is of extremely importance. The detection of 25-hydroxyVitamin-D3 and other bone metabolism indexes contributes to the evaluation of the progress of the disease and bone metabolism in patients with GDM.

### Limitations:

The number of subjects included in this study is limited, so the conclusions drawn may not be very convincing. For example, there was no statistically significant difference in blood calcium level between the two groups in this study, which may be due to the small sample size. In addition, given that the study was a small retrospective study, these results are not conclusive. Further large-scale trials are needed to confirm our observations.

## CONCLUSION

The BMD and 25-hydroxyvitamin levels of patients with GDM are lower than those of normal pregnant women. The higher the blood glucose of pregnant women, the more severe their Vitamin-D deficiency and the lower the BMD. In view of this, while treating pregnant women with GDM, Vitamin-D should be actively supplemented to improve BMD, so as to protect maternal and infant health.

### Authors’ Contributions:

**LH &** **JM:** Designed this study and prepared this manuscript, and are responsible and accountable for the accuracy or integrity of the work.

**ZL:** Collected and analyzed clinical data.

**SW:** Significantly revised this manuscript.
